# Supplementary material for: Effort produces after-effects costly for others but valued for self
Source: eLife. 2026 May 14;13:RP103566. doi: 10.7554/eLife.103566 (PMC13175574; doi:10.7554/eLife.103566)
Supplement: Supplementary file 1. [file elife-103566-supp1.docx]

**Supplementary file 1.** Results of mixed-effects models predicting response success (logistic; left) and response speed (linear; right) in the prosocial effort task

|  | Response success | | | Response speed | | |
| --- | --- | --- | --- | --- | --- | --- |
| Predictors | *b* | 95% CI | *p* | *b* | 95% CI | *p* |
| Intercept | 8.71 | 6.79, 10.63 | **<0.001** | 5.15 | 4.91, 5.39 | **<0.001** |
| Recipient (R) | -0.46 | -2.34, 1.42 | 0.632 | -0.07 | -0.11, -0.04 | **<0.001** |
| Effort (E) | -4.77 | -6.14, -3.41 | **<0.001** | 0.56 | 0.47, 0.64 | **<0.001** |
| Magnitude (M) | 0.45 | -0.31, 1.20 | 0.246 | 0.01 | -0.00, 0.02 | 0.128 |
| R:E | 0.07 | -1.29, 1.42 | 0.923 | 0.02 | -0.00, 0.05 | 0.074 |
| R:M | 0.40 | -1.11, 1.90 | 0.606 | -0.00 | -0.03, 0.02 | 0.847 |
| E:M | -0.24 | -0.79, 0.31 | 0.400 | 0.01 | -0.01, 0.02 | 0.276 |
| R:E:M | -0.18 | -1.28, 0.92 | 0.748 | 0.00 | -0.03, 0.03 | 0.965 |
| Observations | 8000 |  |  | 8000 |  |  |

*Notes*: The final model for response success data was specified as Response success ~ Recipient * Effort * Magnitude + (Recipient + Effort | Participant) and for response speed data as: Response speed ~ Recipient * Effort * Magnitude + (Recipient + Effort | Participant). Both effort and magnitude levels were standardized before being entered into the model. For response success, *b* values are expressed in log-odds units from a logistic mixed-effects model, whereas for response speed, *b* values are estimates from a linear mixed-effects model. Statistically significant *p* values (< 0.05, two-sided) are shown in bold. CI = confidence interval.
