# Supplementary material for: Effort produces after-effects costly for others but valued for self
Source: eLife. 2026 May 14;13:RP103566. doi: 10.7554/eLife.103566 (PMC13175574; doi:10.7554/eLife.103566)
Supplement: Supplementary file 2. [file elife-103566-supp2.docx]

**Supplementary file 2.** Results of linear regression models predicting rating data of difficulty, effort, and liking

|  | Difficulty | | | Effort | | | Liking | | |
| --- | --- | --- | --- | --- | --- | --- | --- | --- | --- |
| Predictors | *b* | 95% CI | *p* | *b* | 95% CI | *p* | *b* | 95% CI | *p* |
| Intercept | 3.96 | 3.66, 4.25 | **<0.001** | 5.17 | 4.63, 5.70 | **<0.001** | 6.39 | 6.00, 6.78 | **<0.001** |
| Recipient (R) | 0.19 | -0.14, 0.51 | 0.260 | -0.32 | -0.58, -0.06 | **0.019** | -0.62 | -0.98, -0.26 | **0.001** |
| Effort (E) | 2.38 | 2.19, 2.57 | **<0.001** | 1.90 | 1.54, 2.26 | **<0.001** | -1.79 | -2.11, -1.46 | **<0.001** |
| R:E | 0.07 | -0.16, 0.30 | 0.548 | -0.11 | -0.30, 0.09 | 0.270 | **-**0.28 | -0.51, -0.04 | **0.023** |
| Observations | 400 |  |  | 400 |  |  | 400 |  |  |

*Notes*. The final model for rating data was specified as: Rating ~ Recipient * Effort + (Recipient + Effort | Participant). Effort level was standardized before being entered into the model. Statistically significant *P* values (< 0.05, two-sided) are shown in bold. CI = confidence interval.
