# Supplementary material for: Effort produces after-effects costly for others but valued for self
Source: eLife. 2026 May 14;13:RP103566. doi: 10.7554/eLife.103566 (PMC13175574; doi:10.7554/eLife.103566)
Supplement: Supplementary file 3. [file elife-103566-supp3.docx]

**Supplementary file 3.** Results of RewP models with response speed (left) and effort rating (right) as covariates

|  | RewP (including response speed) | | | RewP (including effort rating) | | |
| --- | --- | --- | --- | --- | --- | --- |
| Predictor | *b* | 95% CI | *p* | *b* | 95% CI | *p* |
| Intercept | 2.94 | 1.93, 3.95 | **<0.001** | 2.42 | 1.16, 3.68 | **<0.001** |
| Recipient (R) | -0.69 | -1.20, -0.18 | **0.011** | -0.65 | -1.17, -0.13 | **0.015** |
| Effort (E) | -0.01 | -0.32, 0.29 | 0.923 | -0.25 | -0.63, 0.12 | 0.187 |
| Magnitude (M) | 0.42 | 0.15, 0.70 | **0.003** | 0.42 | 0.15, 0.70 | **0.003** |
| Valence (V) | -1.08 | -1.49, -0.68 | **<0.001** | -1.08 | -1.49, -0.68 | **<0.001** |
| Speed/Effort rating | -0.07 | -0.34, 0.20 | 0.614 | 0.10 | -0.05, 0.25 | 0.186 |
| R:E | -0.55 | -0.96, -0.14 | **0.009** | -0.54 | -0.95, -0.13 | **0.010** |
| R:M | -0.39 | -0.79, 0.02 | 0.062 | -0.38 | -0.79, 0.02 | 0.063 |
| E:M | -0.04 | -0.25, 0.16 | 0.687 | -0.04 | -0.25, 0.16 | 0.688 |
| R:V | 0.59 | -0.22, 1.40 | 0.152 | 0.59 | -0.22, 1.40 | 0.153 |
| E:V | -0.19 | -0.60, 0.22 | 0.371 | -0.19 | -0.60, 0.23 | 0.375 |
| M:V | 0.13 | -0.28, 0.53 | 0.544 | 0.13 | -0.28, 0.53 | 0.545 |
| R:E:M | -0.49 | -0.90, -0.08 | **0.019** | -0.49 | -0.90, -0.08 | **0.019** |
| R:E:V | 0.69 | -0.13, 1.51 | 0.099 | 0.69 | -0.13, 1.51 | 0.100 |
| R:M:V | 0.86 | 0.05, 1.66 | **0.038** | 0.86 | 0.05, 1.67 | **0.038** |
| E:M:V | 0.07 | -0.34, 0.49 | 0.725 | 0.07 | -0.34, 0.49 | 0.722 |
| R:E:M:V | 0.69 | -0.14, 1.51 | 0.103 | 0.68 | -0.14, 1.51 | 0.105 |
| Observations | 7566 |  |  | 7566 |  |  |

*Notes*. The final model was specified as: Amplitude ~ Recipient * Effort * Magnitude * Valence + (Recipient + Effort + Magnitude | Participant). Response speed was standardized before modeling. However, effort ratings were centered rather than standardized, as three participants showed no variance in their ratings (reporting the same value across all levels), which would have caused standardization to yield NaN values.
