# Supplementary material for: Effort produces after-effects costly for others but valued for self
Source: eLife. 2026 May 14;13:RP103566. doi: 10.7554/eLife.103566 (PMC13175574; doi:10.7554/eLife.103566)
Supplement: Supplementary file 4. [file elife-103566-supp4.docx]

**Supplementary file 4.** Results of a linear mixed-effects model predicting P3 amplitudes in response to performance feedback in the prosocial effort task

| Predictor | *b* | 95% CI | *p* |
| --- | --- | --- | --- |
| Intercept | 1.31 | 0.74, 1.88 | **<0.001** |
| Recipient (R) | -0.15 | -0.48, 0.19 | 0.393 |
| Effort (E) | 0.72 | 0.45, 0.99 | **<0.001** |
| Magnitude (M) | 0.01 | -0.16, 0.18 | 0.904 |
| R:E | -0.19 | -0.53, 0.16 | 0.285 |
| R:M  E:M | 0.17  -0.13 | -0.16, 0.51  -0.30, 0.04 | 0.319  0.131 |
| R:E:M | 0.00 | -0.34, 0.34 | 0.984 |
| Observations | 7469 |  |  |

*Notes*. The final model was specified as: Amplitude ~ Recipient * Effort * Magnitude + (Effort | Participant). Both effort and magnitude levels were standardized before being entered into the model. Statistically significant *p* values (< 0.05, two-sided) are shown in bold. CI = confidence interval.
