# Supplementary material for: Effort produces after-effects costly for others but valued for self
Source: eLife. 2026 May 14;13:RP103566. doi: 10.7554/eLife.103566 (PMC13175574; doi:10.7554/eLife.103566)
Supplement: Supplementary file 5. [file elife-103566-supp5.docx]

**Supplementary file 5.** Results of a linear mixed-effects model predicting decision times in the prosocial decision-making task

| Predictors | *b* | 95% CI | *p* |
| --- | --- | --- | --- |
| Intercept | 1470.46 | 1373.63, 1567.29 | **<0.001** |
| Recipient (R) | 37.14 | -10.02, 84.30 | 0.121 |
| Effort (E) | 38.91 | 14.23, 63.59 | **0.003** |
| Effort² (E²) | -64.89 | -90.90, -38.89 | **<0.001** |
| Magnitude (M) | -54.88 | -76.17, -33.59 | **<0.001** |
| R:E | -41.49 | -65.66, -17.31 | **0.001** |
| R:E² | 11.25 | -17.69, 40.19 | 0.446 |
| R:M | 44.86 | 7.00, 82.72 | **0.020** |
| E:M | 33.23 | 21.14, 45.32 | **<0.001** |
| E²:M | 15.14 | 0.66, 29.62 | **0.040** |
| R:E:M | 16.46 | -7.72, 40.64 | 0.182 |
| R:E²:M | -4.42 | -33.38, 24.53 | 0.765 |
| Observations | 5945 |  |  |

*Notes*: The final model was specified as: Decision times ~ Recipient * Effort * Magnitude + Recipient * Effort² * Magnitude + (Recipient + Effort + Effort² + Magnitude | Participant). Both effort and magnitude levels were standardized before being entered into the model. Statistically significant *p* values (< 0.05, two-sided) are shown in bold. CI = confidence interval.
