# Supplementary material for: Effort produces after-effects costly for others but valued for self
Source: eLife. 2026 May 14;13:RP103566. doi: 10.7554/eLife.103566 (PMC13175574; doi:10.7554/eLife.103566)
Supplement: Supplementary file 6. [file elife-103566-supp6.docx]

**Supplementary file 6.** Results of a mixed-effects logistic regression model predicting decision choices in the prosocial decision-making task

| Predictors | *b* | 95% CI | *p* |
| --- | --- | --- | --- |
| Intercept | 2.29 | 1.53, 3.04 | **<0.001** |
| Recipient (R) | -1.74 | -2.23, -1.14 | **<0.001** |
| Effort (E) | -2.54 | -2.93, -2.14 | **<0.001** |
| Magnitude (M) | 2.11 | 1.64, 2.59 | **<0.001** |
| R:E | -0.18 | -0.46, 0.10 | 0.216 |
| R:M | -0.56 | -0.82, -0.31 | **<0.001** |
| E:M | 0.15 | 0.02, 0.29 | **0.028** |
| R:E:M | -0.12 | -0.36, 0.11 | 0.303 |
| Observations | 5945 |  |  |

*Notes*: The final model was specified as: Decision choices ~ Recipient * Effort * Magnitude + (Recipient + Effort + Magnitude | Participant). Both effort and magnitude levels were standardized before being entered into the model. The *b* values are expressed in log-odds units. Statistically significant *p* values (< 0.05, two-sided) are shown in bold. CI = confidence interval.
