# Supplementary material for: Effort produces after-effects costly for others but valued for self
Source: eLife. 2026 May 14;13:RP103566. doi: 10.7554/eLife.103566 (PMC13175574; doi:10.7554/eLife.103566)
Supplement: Supplementary file 7. [file elife-103566-supp7.docx]

**Supplementary file 7.** Results of RewP models with discounting rate (log*K*) and high-effort choice proportions as fixed predictors

|  | log*K* value | | | High-effort choice proportion | | |
| --- | --- | --- | --- | --- | --- | --- |
| Predictor | *b* | 95% CI | *p* | *b* | 95% CI | *p* |
| Intercept | 2.88 | 1.86, 3.90 | **<0.001** | 2.83 | 1.81, 3.86 | **<0.001** |
| Recipient (R) | -0.45 | -1.09, 0.19 | 0.166 | -0.42 | -1.04, 0.20 | 0.180 |
| Effort (E) | 0.00 | -0.27, 0.27 | 0.990 | 0.00 | -0.26, 0.27 | 0.975 |
| Magnitude (M) | 0.42 | 0.13, 0.72 | **0.006** | 0.40 | 0.12, 0.69 | **0.007** |
| Valence (V) | -1.16 | -1.60, -0.71 | **<0.001** | -1.10 | -1.54, -0.67 | **<0.001** |
| logK (K)/Choice (C) | -0.35 | -0.85, 0.15 | 0.163 | 0.36 | -0.14, 0.87 | 0.153 |
| R:E | -0.72 | -1.18, -0.26 | **0.002** | -0.73 | -1.18, -0.27 | **0.002** |
| R:M | -0.41 | -0.87, -0.04 | 0.077 | -0.32 | -0.77, 0.13 | 0.160 |
| E:M | -0.16 | -0.38, 0.07 | 0.177 | -0.15 | -0.37, 0.08 | 0.197 |
| R:V | 0.20 | -0.68, 1.09 | 0.655 | 0.45 | -0.42, 1.32 | 0.307 |
| E:V | -0.37 | -0.82, 0.08 | 0.106 | -0.34 | -0.78, 0.10 | 0.134 |
| M:V | 0.18 | -0.26, 0.63 | 0.418 | 0.20 | -0.23, 0.64 | 0.361 |
| R:K/C | 0.41 | -0.21, 1.03 | 0.185 | -0.60 | -1.22, 0.02 | 0.057 |
| E:K/C | 0.19 | -0.07, 0.45 | 0.144 | -0.23 | -0.49, 0.03 | 0.077 |
| M:K/C | 0.02 | -0.25, 0.29 | 0.875 | 0.08 | -0.19, 0.34 | 0.573 |
| V:K/C | 0.44 | 0.00, 0.89 | **0.050** | -0.21 | -0.65, 0.24 | 0.363 |
| R:E:M | -0.39 | -0.84, 0.06 | 0.093 | -0.41 | -0.85, 0.04 | 0.072 |
| R:E:V | 0.47 | -0.43, 1.37 | 0.307 | 0.53 | -0.35, 1.42 | 0.240 |
| R:M:V | 1.26 | 0.37, 2.14 | **0.005** | 1.13 | 0.26, 2.00 | **0.011** |
| E:M:V | -0.09 | -0.54, 0.37 | 0.710 | 0.01 | -0.44, 0.45 | 0.979 |
| R:E:K/C | -0.27 | -0.74, 0.20 | 0.262 | 0.34 | -0.12, 0.81 | 0.149 |
| R:M:K/C | 0.01 | -0.46, 0.48 | 0.973 | -0.13 | -0.60, 0.34 | 0.594 |
| E:M:K/C | -0.08 | -0.31, 0.14 | 0.471 | 0.10 | -0.13, 0.33 | 0.380 |
| R:V:K/C | 0.10 | -0.79, 0.99 | 0.820 | -0.14 | -1.04, 0.75 | 0.752 |
| E:V:K/C | 0.34 | -0.11, 0.80 | 0.139 | -0.25 | -0.71, 0.20 | 0.272 |
| M:V:K/C | -0.43 | -0.87, 0.02 | 0.060 | 0.38 | -0.07, 0.83 | 0.094 |
| R:E:M:V | 0.43 | -0.47, 1.33 | 0.350 | 0.58 | -0.31, 1.46 | 0.203 |
| R:E:M:K/C | 0.58 | 0.12, 1.03 | **0.013** | -0.58 | -1.04, -0.12 | **0.014** |
| R:E:V:K/C | 0.82 | -0.09, 1.72 | 0.077 | -0.86 | -1.78, 0.05 | 0.063 |
| R:M:V:K/C | -0.66 | -1.56, 0.23 | 0.145 | 0.44 | -0.46, 1.33 | 0.341 |
| E:M:V:K/C | 0.06 | -0.40, 0.52 | 0.794 | -0.13 | -0.59, 0.33 | 0.581 |
| R:E:M:V:K/C | 0.71 | -0.20, 1.62 | 0.128 | -0.39 | -1.31, 0.53 | 0.406 |
| Observations | 7375 |  |  | 7566 |  |  |

*Notes*. The final model was specified as: Amplitude ~ Recipient * Effort * Magnitude * Valence * log*K*/Choice + (Recipient + Effort + Magnitude | Participant). Higher log*K* values are associated with lower high-effort choice proportions.
