# Supplementary material for: Effort produces after-effects costly for others but valued for self
Source: eLife. 2026 May 14;13:RP103566. doi: 10.7554/eLife.103566 (PMC13175574; doi:10.7554/eLife.103566)
Supplement: Supplementary file 8. [file elife-103566-supp8.docx]

**Supplementary file 8.** Simulation-based sensitivity analysis for fixed effects in the RewP model

| Regression equation | Tested effect | *p* | Original effect  (*b* ± *SE*) | Smallest effect  (*b*) |
| --- | --- | --- | --- | --- |
| RewP ~ Recipient × Effort × Magnitude × Valence | Recipient (R) | **0.012** | -0.68 ± 0.26 | -0.73 |
|  | Effort (E) | 0.624 | -0.06 ± 0.12 | -0.34 |
|  | Magnitude (M) | **0.003** | 0.42 ± 0.14 | 0.39 |
|  | Valence (V) | **<0.001** | -1.08 ± 0.21 | -0.56 |
|  | R:E | **0.009** | -0.55 ± 0.21 | -0.55 |
|  | R:M | 0.062 | -0.39 ± 0.21 | -0.55 |
|  | E:M | 0.685 | -0.04 ± 0.11 | -0.28 |
|  | R:V | 0.153 | 0.59 ± 0.41 | 1.12 |
|  | E:V | 0.372 | -0.19 ± 0.21 | -0.57 |
|  | M:V | 0.546 | 0.12 ± 0.21 | 0.57 |
|  | R:E:M | **0.019** | -0.49 ± 0.21 | -0.56 |
|  | R:E:V | 0.099 | 0.69 ± 0.42 | 1.11 |
|  | R:M:V | **0.038** | 0.86 ± 0.41 | 1.12 |
|  | E:M:V | 0.721 | 0.08 ± 0.21 | 0.56 |
|  | R:E:M:V | 0.105 | 0.68 ± 0.42 | 1.10 |

*Notes*. The smallest effect size refers to the minimum detectable unstandardized regression coefficient (*b*) detectable with 80% power. For simplicity, only the fixed-effect structure is displayed in the regression equation column. Statistically significant *p* values (< .05, two-sided) are shown in bold. *SE* = standard error.
